# Supplementary material for: Novel Driver Strength Index highlights important cancer genes in TCGA PanCanAtlas patients
Source: PeerJ. 2022 Aug 11;10:e13860. doi: 10.7717/peerj.13860 (PMC9375969; doi:10.7717/peerj.13860)
Supplement: Supplemental Information 2 [file peerj-10-13860-s002.zip › COHORTS/genes plots/detailed/2021_8_16_14_9_distribution_events_detailed_7.pdf]

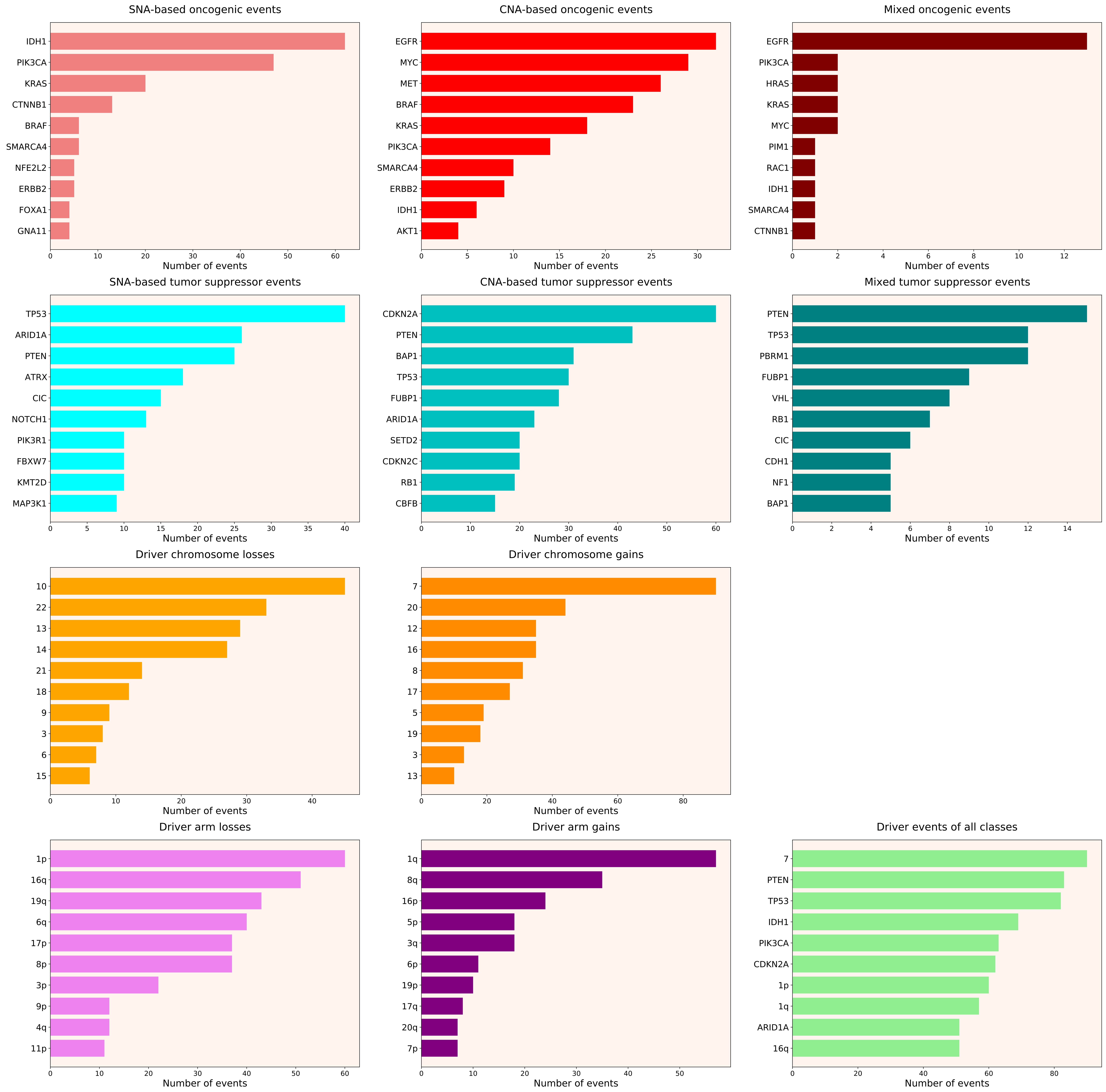
SNA-based oncogenic events
IDH1
PIK3CA
KRAS
CTNNB1
BRAF
SMARCA4
NFE2L2
ERBB2
FOXA1
GNA11
Number of events
CNA-based oncogenic events
EGFR
MYC
MET
BRAF
KRAS
PIK3CA
SMARCA4
ERBB2
IDH1
AKT1
Number of events
Mixed oncogenic events
EGFR
PIK3CA
HRAS
KRAS
MYC
PIM1
RAC1
IDH1
SMARCA4
CTNNB1
Number of events
SNA-based tumor suppressor events
TP53
ARID1A
PTEN
ATRX
CIC
NOTCH1
PIK3R1
FBXW7
KMT2D
MAP3K1
Number of events
CNA-based tumor suppressor events
CDKN2A
PTEN
BAP1
TP53
FUBP1
ARID1A
SETD2
CDKN2C
RB1
CBFB
Number of events
Mixed tumor suppressor events
PTEN
TP53
PBRM1
FUBP1
VHL
RB1
CIC
CDH1
NF1
BAP1
Number of events
Driver chromosome losses
10
22
13
14
21
18
9
3
6
15
Number of events
Driver chromosome gains
7
20
12
16
8
17
5
19
3
13
Number of events
Driver arm losses
1p
16q
19q
6q
17p
8p
3p
9p
4q
11p
Number of events
Driver arm gains
1q
8q
16p
5p
3q
6p
19p
17q
20q
7p
Number of events
Driver events of all classes
7
PTEN
TP53
IDH1
PIK3CA
CDKN2A
1p
1q
ARID1A
16q
Number of events
